# Identification of knee gait waveform pattern alterations in individuals with patellofemoral pain using fast Fourier transform

**DOI:** 10.1371/journal.pone.0209015

**Published:** 2018-12-14

**Authors:** Kristin D. Morgan, Brian Noehren

**Affiliations:** 1 Biomedical Engineering, School of Engineering, University of Connecticut, Storrs, CT, United States of America; 2 Division of Physical Therapy, College of Health Sciences, University of Kentucky, Lexington, KY, United States of America; Holland Bloorview Kids Rehabilitation Hospital, CANADA

## Abstract

Patellofemoral pain (PFP) is one of the most common overuse injuries of the knee. Previous research has found that individuals with PFP exhibit differences in peak hip kinematics; however, differences in peak knee kinematics, where the pain originates, are difficult to elucidate. To better understand the mechanism behind PFP, we sought to characterize differences in knee gait kinematic waveform patterns in individuals with PFP compared to healthy individuals using fast Fourier transform (FFT). Sixteen control and sixteen individuals with PFP participated in a fast walk protocol. FFT was used to decompose the sagittal, frontal and transverse plane knee gait waveforms into sinusoidal signals. A two-way ANOVA and Bonferroni post hoc analysis compared group, limb and interaction effects on sagittal, frontal and transverse amplitude, frequency and phase components between control and PFP individuals gait waveforms. Differences in frequency and phase values were found in the sagittal and frontal plane knee waveforms between the control and PFP groups. The signal-to-noise ratio also reported significant differences between the PFP and control limbs in the sagittal (p<0.01) and frontal planes (p = 0.04). The findings indicate that differences in gait patterns in the individuals with PFP were not the result of amplitude differences, but differences attributed to temporal changes in gait patterns detected by the frequency and phase metrics. These changes suggest that individuals with PFP adopted a more deliberate, stiffer gait and exhibit altered joint coordination. And the FFT technique could serve as a fast, quantifiable tool for clinicians to detect PFP.

## Introduction

Patellofemoral pain (PFP) is one of the most common overuse running injuries in active populations; such as runners and the military, and is known to affect women at a disproportionately higher rate than men [1–3]. PFP often becomes chronic with up to 50–90% of individuals reporting continued symptoms at 4–19 year follow ups [4–6]. Alterations in joint mechanics in subjects with PFP have most commonly been observed at the hip based on analysis of peak kinematics during tasks such as walking and running; despite PFP pain originating at the knee [7,8]. The difficulty in detecting differences at the knee suggests that alternate techniques could be explored to capture changes in knee gait mechanics in individuals with PFP. Previous research found that individuals suffering from PFP are characterized by delayed quadriceps muscle activation but not deficits in muscle strength [9–12]. This altered muscle activation could explain why differences in peak knee angle are not always present but differences in the timing of peak knee angles may be. Fast Fourier Transform (FFT) is a technique that can assess both magnitude and temporal characteristics in waveforms and has successfully identified critical temporal metrics from gait data in individuals with neurological disorders [13,14]. Here FFT was used to identify both magnitude and temporal differences in knee kinematics in control individuals and those suffering from PFP during fast walking.

FFT is an established method that has not been extensively explored in individuals with PFP [13–18]. Tinley et al. (2002) [18] used Fourier analysis to develop a gait index to classify abnormal gait in children from sagittal plane hip, knee and ankle kinematic waveforms. Schneider et al. (1983) [19] used it to delineate differences in individual’s knee joint disease from their ground reaction force (GRF) gait patterns. Both studies focused on detecting magnitude differences in gait patterns. Winfree et al. (2015) [20], however, were able to extract temporal differences in stride patterns in individuals with neurological impairments. The ability to assess gait quality and detect joint degeneration from gait patterns highlights the sensitivity of the FFT method and suggests that FFT could identify differences in knee kinematics that may be present in individuals with PFP.

Gait variability is often used to identify the presence of a pathological condition [21–24]. Cunningham et al. (2014) [21] found that individuals with PFP exhibited greater variability at specific intervals during the gait cycle, which they suggested represented a deliberate act to alter loading on the knee. Conversely, similar studies have reported reduced variability in individuals with PFP when assessing coupling angle variability [22,23]. This study used signal-to-noise ratio to quantify knee kinematic variability during fast walking. Given that each participant performed the same walking task, they each will exhibit similar knee kinematic “signals.” Therefore, differences in amplitude, frequency and phase obtained from the FFT analysis will reflect the signal “noise.” While SNR (signal-to-noise ratio) is typically used for signal and image filtering, we will explore its ability to denote gait variability in individuals with PFP [25,26].

The objective of this paper is to implement FFT to detect changes in sagittal, frontal, and transverse knee gait patterns during fast walking from the amplitude, frequency and phase components in controls and individuals with PFP. Additionally, SNR will be used to assess gait pattern variability between the two populations. We hypothesize that individuals suffering from PFP will not show any differences in gait amplitude but will exhibit larger frequency and phase values which would reflect delayed peak knee angles during stance. We also hypothesize that the PFP individuals will exhibit greater knee gait kinematic variability based on SNR than their control counterparts. Differences observed in these metrics could serve as gait biomarkers for the early identification of PFP.

## Methods

### Instrumented gait analysis

The University of Kentucky Institutional Review Board granted permission to conduct this research. The participants ranged from 18 to 35 years old and provided written informed consent required by the institutional review board. Sixteen control (mean (SD) age: 21.1 (3.9) yrs; height: 1.7 (0.1) m; mass: 64.5 (11.8) kg) and sixteen PFP (age: 22.4 (4.0) yrs; height: 1.7 (0.1) m; mass: 71.4 (19.5) kg) participants performed a fast walking protocol. A power analysis indicated that at least of 15 participants per group were needed to detected a moderate effect (0.75) to achieve adequate statistical power (α = 0.05; 1-β = 0.80). PFP was verified in the participants by a licensed physical therapist. The physical therapist confirmed the individual had PFP if they reported a minimum pain of 3 out of 10 in their patellofemoral joint on the numerical pain rating scale when running and they had to have pain during either the patellar compression or retropatellar/peripatellar palpation test. Physical examination eliminated ligamentous, tendon and internal derangement. Participants were excluded if they had any previous patellar dislocation and/or instability. All control participants could not have had a previous knee surgery and were injury free for the past six months. The control group was age, weight and height matched to the PFP group.

An established fifty-six reflective marker design was used to place anatomical and tracking markers on the participants [8,24]. Anatomical markers were positioned on the C7 vertebrae, acromion processes, sternum located on the trunk, the iliac spines, iliac crests, greater trochanters, femoral epicondyles, tibial plateaus, malleoli, and the first and fifth metatarsal heads on the lower extremities. Four sets of four rigid cluster markers were placed on the thighs and shanks and they served as the tracking markers during the walking trials. The clusters are rigid shells with four markers on them that were tightly wrapped on the distal end of the shank and thigh of the individual. This approach of including clusters was used to minimize the effect of soft tissue artifact and Manal et al. (2003) [27] previously showed this had good agreement with the results using bone pins. For this study, each participant walked on a Bertec instrumented treadmill (Bertec Corp, Columbus, Ohio; New Balance, Brighton, MA) with embedded force plates while wearing WR662 running sneakers. Prior to the data collection, each participant also performed a five-minute walking warm-up to become acclimated to the treadmill. To determine the fast walk speed, participants walked on the treadmill as the speed was repeatedly increased by 0.1m/s. When the speed increased to the point the participants could no longer walk but had to run, the speed was decreased by 0.1m/s and set as the fast walk speed. Participants performed a fast walking protocol because at slower speeds individuals may be able to compensate for the pain. However, because faster speeds require greater muscle activation, any differences in motor control should be magnified [28,29]. Additionally, running has completely different task demands and is not well tolerated by all individuals with PFP. Ten consecutive strides were collected during the fast walk for each individual. Marker trajectories were recorded via a 10-camera motion capture system (Motion Analysis Corp, Santa Rosa, USA) and sampled at 200 Hz. Visual 3D (C-motion, Germantown, MD, USA) was used to filter the marker data, compute the functional hip joint centers and perform inverse kinematics for the lower extremities. The knee joint center was obtained by computing the midpoint of the femoral markers [27]. The marker data was low-pass filtered at 8Hz using a 4^th^ order Butterworth filter and joint angles were resolved using the X-Y-Z Cardan angle sequence referencing the distal with respect to the proximal.

### Spectral analysis of gait kinematics

Ten gait strides were analyzed for each limb, in each plane for the FFT analysis. The FFT converted the sagittal, frontal and transverse plane knee kinematics from the time to the frequency domain. The FFT converted the sagittal, frontal and transverse waveforms from the time to frequency domain by representing the waveforms as a summation of sinusoids [16]. Sinusoidal functions have amplitude, frequency and phase components. The waveforms being represented by multiple sinusoids will have multiple amplitude, frequency and phase components for each sinusoid representing the gait waveform.

We adopted a well-established method for FFT and power spectrum generation [30,31]. Prior to the FFT analysis the data was padded from 2000 points which is approximately 10 seconds of data to 2048 points. Then the FFT was performed and power and phase spectra were produced from the resulting data. Due to padding the data, we matched the power of the power spectrum to that of the power of the original gait waveform for each frequency [32]. For the spectra the frequencies ranged from 0 to half of the sampling rate and they were normalized to 1Hz bins.

The three largest amplitudes obtained from the power spectrum represented the three dominant sinusoidal signals in the gait waveform. The three frequencies at which these dominant amplitudes occurred were stored as they represent the frequency component of the respective sinusoidal functions. The phases for the sinusoids were identified from the phase spectrum. Like the power spectrum, the phase spectrum represents the phase as a function of frequency. To determine the phases, we identified the phases at the same frequencies as the dominant amplitudes. Thus if the power spectrum had a dominant peak of 31 (Deg^2^/Hz) at frequency 1.5Hz, the accompanying phase would be the phase at 1.5Hz. Phase values range from 0 to 360°; however, here phases were normalized to range from 0° to 180° by reducing the values in half. Since this was applied to all phase values it did not affect the results. The FFT analysis was performed using a custom MATLAB program (MATLAB 8.0, The MathWorks, Inc., Natick, Massachusetts, USA).

**Fig 1:**
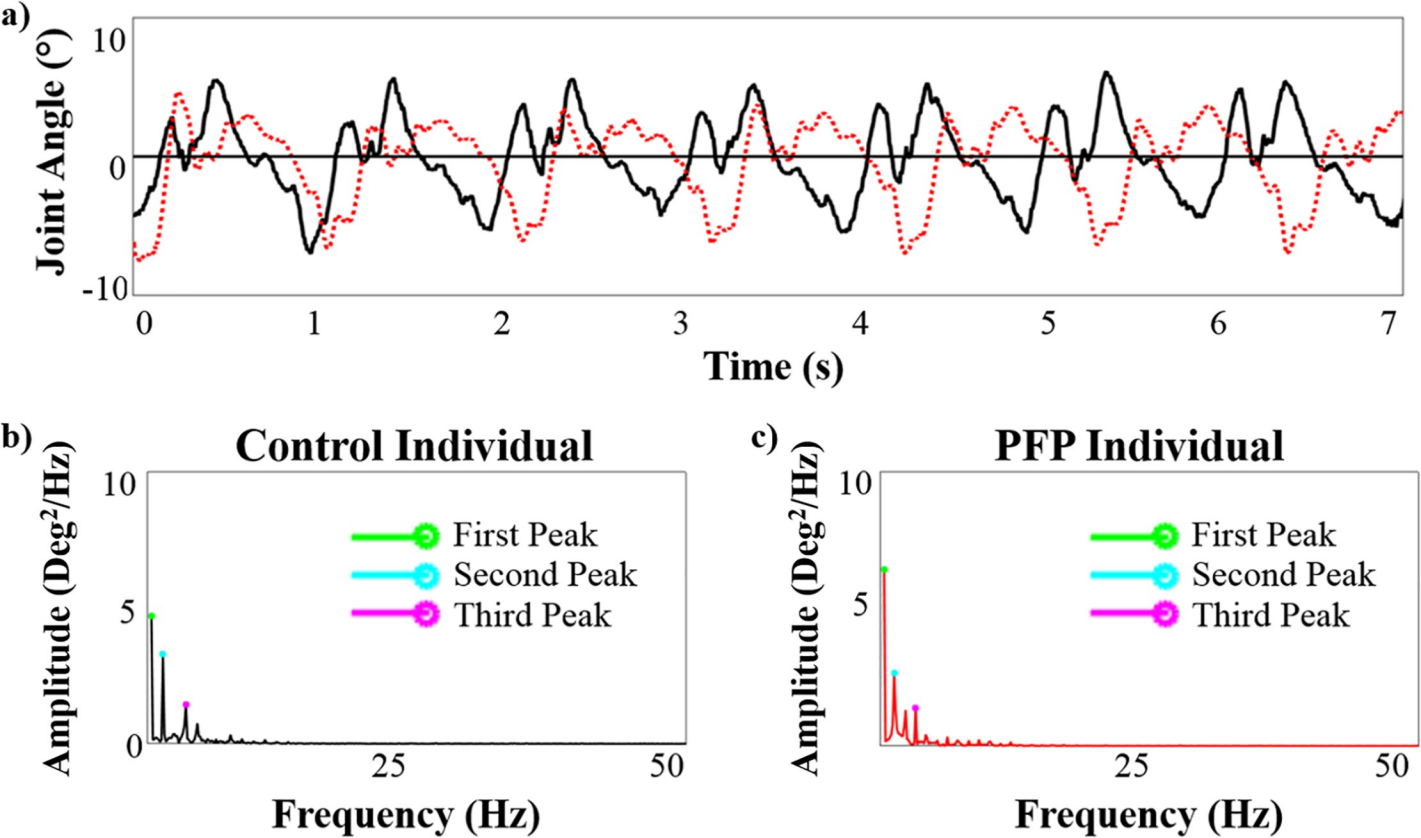
a) Frontal plane knee fast walking kinematic waveforms for control (solid black line) and PFP (dotted red line) individuals. Power spectrums for the (b) control and (c) PFP individuals. The top three amplitudes for the power spectra are denoted and represent the three signals that were used for analysis.

Similar to Lamoth et al. (2002) [33], we used the power spectrum results to determine the cutoff for the number of waveforms analyzed. The first three waveforms were analyzed because they contained 90% of the signal energy (Fig 1). Due to the reduced magnitude, any possible differences observed in that waveform would have had minimal effect on the waveform compared to those observed in the first three waveforms.

### Gait waveform variability evaluation

The first step in the signal-to-noise ratio (SNR) was to subtract the dominant sinusoid (sinusoid 1) from the knee kinematic waveform and becoming the noise signal. The unmodified knee kinematic waveform is the original signal. The SNR computes the ratio of the powers (P) for the original and noise signals, where x is the signal and t is time (Eqs 1–2) [34].

$${SNR}_{dB}=10{log}_{10}\left(\frac{P_{signal}}{P_{noise}}\right)$$ Eq 1

$$\mathrm{where}\;P=\frac{1}{T}\sum_{t=1}^{T}{x(t)}^{2}$$ Eq 2

The result of this ratio is provided in decibels (dB). Lower SNRs indicate greater variability in the signal.

### Patellofemoral pain and control waveform component analysis comparison

**Fig 2:**
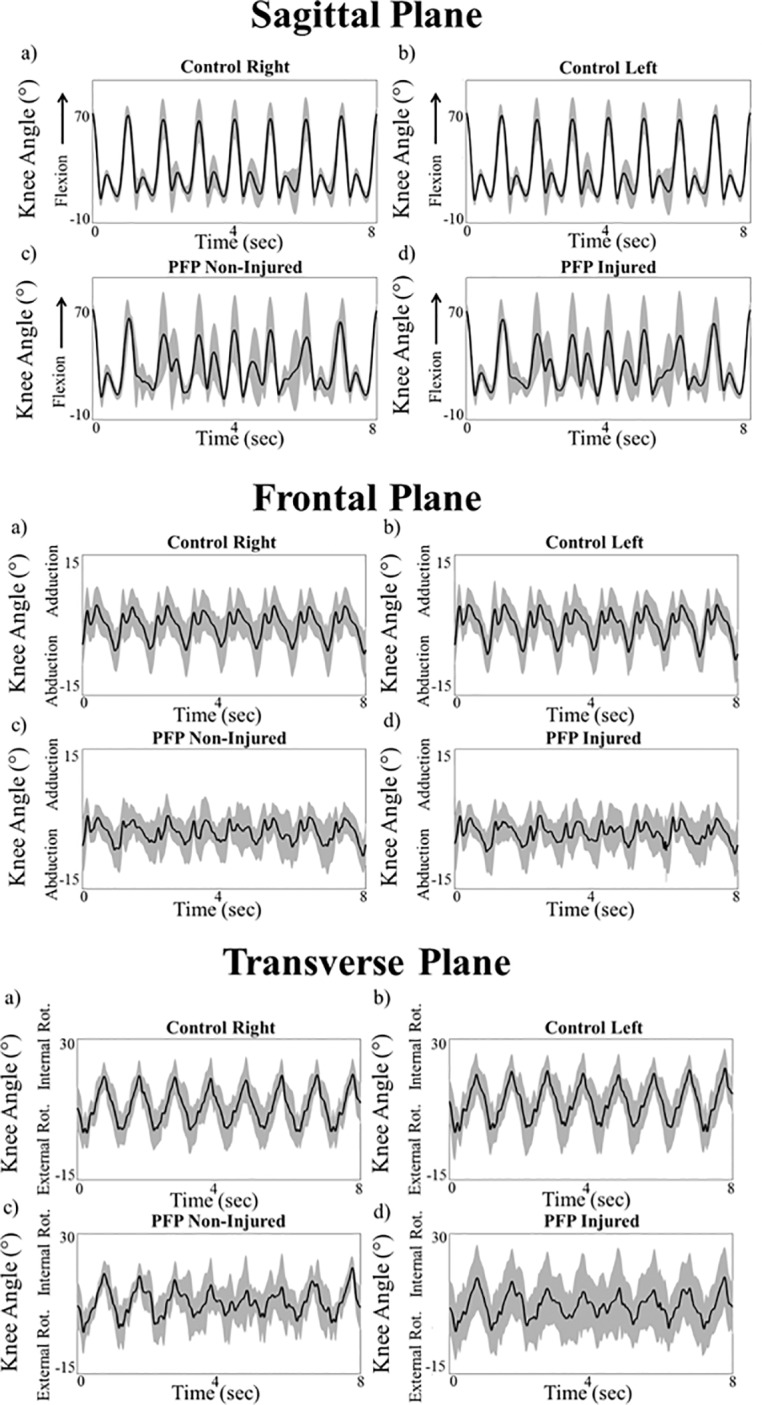
Comparison of the group curves for sagittal, frontal and transverse plane knee fast walking kinematics for the control and PFP individuals. The solid line represents the mean kinematic waveform and the shaded region is the standard deviation. The control right (a) and left limbs (b), are in the top row and the PFP non-injured (c) and injured (d) limbs are in the bottom row.

Ensemble curves of the sagittal, frontal and transverse plane knee kinematic waveforms were generated for the control and PFP groups (Fig 2). The Anderson-Darling Test and Bartlett’s and Levene’s tests were performed to test the data normality and the equality of the variances, respectively. A two-way ANOVA with a Bonferroni post hoc analysis was performed to compare the main effects of group and limb and the interaction of group and limb on the sagittal, frontal and transverse plane amplitude, frequency and phase measures (α = 0.05). A one-way ANOVA and Bonferroni post hoc analysis was conducted on the SNR data to evaluate between limbs differences in SNR values in the sagittal, frontal and transverse planes (α = 0.05). All statistical analyses were conducted in SPSS (Version 23, IBM, Amonk, NY, USA.).

## Results

The comparison of the control and PFP group’s age, mass, height and walking speed reported no between group differences for any of these metrics (Table 1). In the sagittal plane, between group differences were found in the frequency for the second sinusoid signals (p = 0.02) and between limb differences in the PFP group were detected in the phase for the first sinusoidal signal (p = 0.01) (Table 2). The frequency values were significantly smaller in the PFP group compared to the control group. The phase values for the PFP injured limb were significantly larger than the non-injured limb.

**Table 1:** Comparison of mean control and patellofemoral pain participant demographics.

	Control	PFP	P-Value
**Age (yrs)**	21.1 (3.9)	22.4 (4.0)	0.34
**Mass (kg)**	64.5 (11.8)	71.4 (19.5)	0.24
**Height (m)**	1.7 (0.1)	1.7 (0.1)	0.89
**BMI**	22.7 (4.1)	24.6 (3.8)	0.18
**Walk Speed (m/s)**	1.9 (0.1)	1.9 (0.2)	0.84

**Table 2:** Comparison of the three dominant sinusoid amplitudes, frequencies and phases in the sagittal, frontal and transverse planes for the control and patellofemoral pain limbs.

**AMPLITUDE**						
**Direction**	**Sinusoid**	**Control Right**	**Control** **Left**	**PFP** **Non-Injured**	**PFP** **Injured**	**P-Value**
**Sagittal (Deg**^**2**^**/Hz)**	**1**	22.9 (1.9)	23.0 (2.6)	22.6 (3.0)	22.6 (2.7)	0.97
	**2**	19.1 (1.7)	18.8 (2.5)	19.1 (1.6)	18.0 (2.8)	0.47
	**3**	5.3 (1.6)	5.8 (2.6)	6.3 (2.7)	6.0 (2.2)	0.67
**Frontal (Deg**^**2**^**/Hz)**	**1**	4.4 (1.2)	4.6 (1.3)	4.3 (1.6)	4.4 (1.8)	0.97
	**2**	2.7 (1.1)	2.7 (0.7)	2.7 (0.7)	2.7 (1.2)	0.99
	**3**	1.8 (0.7)	1.9 (0.6)	1.8 (0.6)	1.8 (0.7)	0.95
**Transverse (Deg**^**2**^**/Hz)**	**1**	8.9 (2.4)	9.4 (3.0)	8.2 (2.1)	9.8 (7.1)	0.70
	**2**	6.1 (2.3)	4.7 (2.3)	6.2 (2.4)	5.5 (2.0)	0.24
	**3**	2.3 (0.9)	2.2 (0.8)	2.8 (1.4)	2.2 (0.8)	0.36
**FREQUENCY**						
**Direction**	**Sinusoid**	**Control Right**	**Control** **Left**	**PFP** **Non-Injured**	**PFP** **Injured**	**P-Value**
**Sagittal (Hz)**	**1**	1.0 (0.6)	1.0 (0.5)	0.9 (0.3)	1.1 (0.5)	0.34
	**2**	2.0 (0.1)^a^	2.0 (0.1)^a^	2.0 (0.1)	1.8 (0.4)^b^	0.02*
	**3**	3.0 (0.3)	2.9 (0.5)	2.6 (0.8)	2.6 (0.7)	0.25
**Frontal (Hz)**	**1**	0.8 (0.4)	1.0 (0.6)	0.7 (0.6)	0.7 (0.8)	0.37
	**2**	1.2 (1.2)	1.2 (1.1)	1.3 (1.3)	1.0 (0.8)	0.91
	**3**	2.7 (0.5)^a^	2.7 (0.6)^a^	1.7 (1.0)^b^	2.5 (0.8)^a^	0.00*
**Transverse (Hz)**	**1**	0.8 (0.5)	0.8 (0.4)	0.6 (0.5)	0.6 (0.5)	0.32
	**2**	0.7 (1.1)	0.8 (1.3)	0.7 (0.8)	0.8 (1.0)	0.97
	**3**	2.9 (1.1)	2.2 (1.4)	2.9 (1.1)	2.8 (1.0)	0.29
**PHASE**						
**Direction**	**Sinusoid**	**Control Right**	**Control** **Left**	**PFP** **Non-Injured**	**PFP** **Injured**	**P-Value**
**Sagittal (°)**	**1**	50.4 (9.4)	46.3 (10.7)^a^	41.7 (12.7)^a^	60.8 (23.5)^b^	0.00*
	**2**	109.9 (17.9)	112.6 (15.7)	110.1 (14.3)	108.2 (26.8)	0.14
	**3**	129.9 (23.9)	117.8 (42.0)	116.8 (53.2)	118.7 (46.1)	0.80
**Frontal (°)**	**1**	120.5 (63.8)	137.5 (42.7)	107.3 (44.9)	107.9 (48.0)	0.30
	**2**	67.9 (55.6)^a^	72.5 (49.9)^a^	104.8 (48.8)	111.5 (54.1)^b^	0.04*
	**3**	75.4 (29.3)	81.0 (33.7)	65.1 (34.0)	74.6 (31.4)	0.57
**Transverse (°)**	**1**	97.4 (10.6)	91.1 (13.6)	90.7 (7.8)	97.8 (13.1)	0.17
	**2**	94.4 (14.2)	110.9 (50.7)	86.8 (22.8)	95.8 (21.4)	0.17
	**3**	90.1 (61.2)	123.4 (60.2)	101.4 (60.2)	100.4 (61.9)	0.48

*indicates that the group means are significantly different

The letters a and b are used to denote means that are significantly different. Groups that do not share the same letter are significantly different.

In the frontal plane, a group and limb interaction effect was detected in the frequency value for the third sinusoidal signal (p = 0.03) where the PFP group reported lower frequency values than the control group and greater between limb asymmetry (Table 2). Between group differences were detected in the second sinusoidal signal phase value (p<0.01) as both limbs reported significantly larger phase values. The transverse plane did not report any significant differences in amplitude, frequency and phase between the control and PFP groups for any sinusoid.

The SNR analysis found that the PFP injured limb reported significantly lower SNR values than the control right and left limbs in the sagittal plane where the control left limb reported SNR values nearly three times as large as the PFP injured limb (Table 3). In the frontal plane, the PFP non-injured limb reported significantly lower SNR values than the control limbs (p = 0.04). No significant differences were present in the transverse plane.

**Table 3:** Comparison of the signal-to-noise ratio in the sagittal, frontal and transverse planes for control and patellofemoral pain limbs.

Direction	SNR	Control Right	Control Left	PFP Non-Injured	PFP Injured	P-Value
**Sagittal (dB)**		2.4 (1.9)^a^	2.6 (1.6)^a^	1.3 (0.9)	0.9 (1.4)^b^	0.00*
**Frontal (dB)**		-2.0 (1.5)^a^	-2.1 (1.5)^a^	-3.2 (1.3)^b^	-2.0 (1.3)	0.04*
**Transverse (dB)**		-1.8 (0.8)	-2.2 (1.1)	-1.6 (0.7)	-2.2 (1.2)	0.22

*indicates that the group means are significantly different

The letters a and b are used to denote means that are significantly different. Groups that do not share the same letter are significantly different.

## Discussion

The objective of this study was to use FFT to assess differences in knee kinematic waveform patterns between the control and PFP limbs based on their amplitude, frequency and phase components during fast walking. Significant differences in frequency and phase values between the control and PFP groups were found in the sagittal and frontal planes only. Since no differences in gait speed were measured between the groups, the reduced sagittal and frontal plane knee frequency in the PFP limbs suggest an altered, slower movement strategy was adopted by the PFP individuals. Furthermore, the significantly larger phase values suggest that the peak knee flexion-extension and abduction-adduction kinematic events were delayed during the gait cycle. These changes could alter when the knee is loaded or unloaded during the gait cycle and lead to injury [35]. The SNR results reported significantly larger sagittal and frontal variability in the PFP individuals which was consistent with previous work [21]. Thus, the SNR was able to detect the knee kinematic variability contributed by the frequency and phase metrics. Overall the FFT and SNR methods were able to delineate differences in knee kinematics between the control and PFP groups and showed that temporal changes in knee kinematics can contribute to the altered joint loading.

During walking, delayed vastus medialis activation has been repeatedly observed in individuals with PFP [9–12]. This delayed vastus medialis activation contributes to poor tracking of the patella and abnormal joint loading [9,10,36]. The delayed sagittal and frontal plane peak knee kinematics measured from FFT reflects the kinematic changes associated with delayed vastus medialis activation. Sasaki and Neptune (2010) [37] found that even small changes in joint kinematics; largely as a result of quadriceps muscle activation, translated to significant increases in joint loading. Therefore, the subtle changes in joint motion detected by FFT may represent the elevated forces experienced on the knee that are causing pain. Since studies often do not investigate temporal changes in gait kinematics, they would not detect these changes that can have a considerable effect on joint loading [37–39]. The results support the application of FFT and other techniques that investigate both magnitude and temporal differences in joint kinematics in individuals with PFP. These methods can serve as diagnostic tools to help clinicians implement therapeutic techniques that target altering joint kinematic and muscle activation timing for PFP rehabilitation.

Traditionally, SNR is used to detect the presence of noise in a signal to eliminate extraneous information. Here, we found that that extraneous noise was representative of knee kinematic variability. The elevated knee kinematic variability measured using SNR in the PFP group was consistent with work that investigated the temporal component of gait variability in individuals with PFP [21]. This SNR identified that the variability was driven by changes in frequency and phase and showed these changes to be associated with altered joint loading in PFP individuals. While the application of SNR in evaluating movement variability is still new, it appears to be promising and future work will help clarify its clinical significance.

A limitation of this study is the assumption that walking biomechanics are sinusoidal. Walking biomechanics are cyclic and repetitive like sine waves which is why we used FFT to model the knee gait waveforms [32]. Additionally, the FFT amplitude, frequency and phase components are easy to show how these parameters alter waveform pattern. This assumption is found in decades of walking and running biomechanics studies, the results of which have provided important findings about differences in joint and muscle characteristics in different populations and we do not believe this assumption affected the results [30,31,40].

FFT was successfully employed to detect differences in sagittal and frontal plane knee kinematic frequency and phase values in individuals with PFP. These changes revealed that individuals with PFP slowed their knee motion and delayed when the knee was loaded during the gait cycle which resulted in elevated forces experienced at the knee. Previous research identified the significance of timing events in individuals with PFP and this work supports this finding. FFT is a quick and quantifiable method for the detection of individuals with PFP. It provides clinicians with a non-invasive assessment tool to enhance the identification of PFP in individuals. In addition, it can be used to monitor and define rehabilitation protocol(s) needed to speed the individual’s return to previous activity levels.

## Supporting information

S1 File This file contains the data used for the study analysis. (XLSX)
